# Femoral rollback at high-flexion during squatting is related to the improvement of sports activities after bicruciate-stabilized total knee arthroplasty: an observational study

**DOI:** 10.1186/s12891-022-05464-w

**Published:** 2022-05-26

**Authors:** Kenichi Kono, Hiroshi Inui, Tomofumi Kage, Tetsuya Tomita, Takaharu Yamazaki, Shuji Taketomi, Ryota Yamagami, Kohei Kawaguchi, Sakae Tanaka

**Affiliations:** 1 Department of Orthopaedic Surgery, Faculty of Medicine, The University of Tokyo, 7-3-1 Hongo, Bunkyo-ku, Tokyo, 113-0033 Japan; 2 Department of Orthopaedic Biomaterial Science, Osaka University Graduate School of Medicine, Osaka, Japan; 3 Department of Information Systems, Faculty of Engineering, Saitama Institute of Technology, Saitama, Japan

**Keywords:** Bicruciate-stabilized total knee arthroplasty, Sports, Patient-reported outcomes, Kinematics

## Abstract

**Background:**

In bicruciate-stabilized total knee arthroplasty (BCS-TKA), the relationship between the postoperative kinematics and sports subscales in patient-reported outcome measures (PROMs) remains unknown. The purpose of this study was to determine the relationship between kinematics and sports subscales using the PROMs after BCS-TKA.

**Methods:**

Sixty-one patients with severe knee osteoarthritis were examined at 13.5 ± 7.8 months after BCS-TKA. The patients performed squats under single fluoroscopic surveillance in the sagittal plane. Range of motion of the knee, axial rotation of the femur relative to the tibial component, and anteroposterior (AP) translation of the medial and lateral femorotibial contact points were measured using a 2D-to-3D registration technique. In addition, the relationship between the kinematics and improvement of the sports subscales in the Knee Injury and Osteoarthritis Outcome Score (KOOS) was evaluated.

**Results:**

In rotation angle, the femoral external rotation was observed from 0 to 50° of flexion. The amount of femoral external rotation did not correlate with PROMs-SP. In medial AP translation, posterior translation was observed from 0 to 20° and 80–110° of flexion. Mild anterior translation was observed from 20 to 80° of flexion. Beyond 80° of flexion, posterior translation was positively correlated with squatting. In lateral AP translation, posterior translation was observed from 0 to 20° and 80–110° of flexion. Beyond 80° of flexion, posterior translation was positively correlated with running, jumping, twisting/pivoting, and kneeling.

**Conclusion:**

Femoral rollback at high flexion during squatting may be important to improve sports performance after BCS-TKA.

## Background

In bicruciate-stabilized total knee arthroplasty (BCS-TKA), the anterior and posterior cruciate ligaments are substituted, wherein the anatomical implant surface provides physiological kinematics [1]. The Journey II BCS (Smith & Nephew, Memphis, TN, USA) has been redesigned to reduce iliotibial band traction syndrome [2]. The second-generation design is associated with a lower risk of reoperation and revision compared to the first [3]. In addition, BCS-TKA provides acceptable outcomes in terms of activities of daily living (ADL) [1, 4, 5].

Recently, with the improvements in ADL after BCS-TKA, patients have been expected to participate in sports activities. In the Knee Society survey, the number of sports activities allowed after TKA has increased [6]. Furthermore, several studies reported that the return to sports or implant survivorship after TKA is favorable [7–9]. In contrast, Huch et al. reported that the prevalence of postoperative athletic activity was higher in the hip replacement group than in the knee replacement group due to greater pain relief in the hip replacement group [6, 10]. Additionally, Mont et al. reported that the failure rate was higher in the younger and highly active patients [9].

High-flexion activities, such as exercising and gardening, have recently been classified as ADL. Thus, many patients who undergo TKA are expected to perform high-flexion activities. However, it is difficult for the patients to squat, and the limited range of motion (ROM) is negatively correlated with patient expectations [11]. Moreover, Matsushita et al. demonstrated that the improvement in ADL was related to high satisfaction or expectation to be able to perform sports activities [12]. Therefore, the evaluation of patients’ ability to perform high-flexion activities is important.

Several studies have demonstrated that the postoperative knee kinematics was related to the patient-reported outcome measures (PROMs) [13–15]. Van Onsem et al. reported that the high PROM group had more pronounced posterior translation laterally from 60° to 90° of flexion than the low PROM group [16]. However, the relationship between the postoperative kinematics and sports subscales in PROMs remains unknown.

The objective of the current study was to clarify the relationship between kinematics and sports subscales during high-flexion activities based on the PROMs after BCS-TKA. This study hypothesized that posterior translation with flexion was related to the improvement of PROMs concerning sports (PROMs-SP) after BCS-TKA.

## Methods

Sixty-one knees with severe medial osteoarthritis that underwent BCS-TKA were examined. Patients who could perform squatting safely at a natural pace after surgery were examined under single fluoroscopy surveillance in the sagittal plane, according to the previously described method [15]. The indication for BCS-TKA was severe osteoarthritis (OA), with the exception of severe valgus OA (hip-knee-ankle angle > 190°).

To estimate the spatial position and orientation of the femoral and tibial components, a 2D-to-3D registration technique was used [17, 18]. The margin of error of the estimated relative motion between the femoral and tibial components is ≤0.5° for rotation and ≤ 0.4° for translation [17]. The following variables were evaluated: knee flexion, axial rotation of the femoral component relative to the tibial component, and anteroposterior (AP) translation of the medial and lateral femorotibial contact points. A local coordinate system at the femoral component was constructed according to previously described methods [17, 19]. Flexion and femoral external rotation were denoted as positive values. Positive or negative values of AP translation were defined as anterior or posterior to the axes of the tibial component, respectively. The femorotibial contact point was defined as the region in which the proximity of the component surfaces was less than 0.5-mm [15]. All data are expressed as means ± standard deviations.

The Knee Injury and Osteoarthritis Outcome Scores (KOOS) [20] were taken preoperatively (within 1 month) and postoperatively (at the fluoroscopic analyses) as part of PROMs. In addition, the relationship between the kinematics and the improvement of PROMs-SP was evaluated.

All patients provided written informed consent for participation. This study was approved by the concerned institutional review board. In addition, all methods were performed in accordance with the relevant guidelines and regulations.

### Statistical analyses

To analyze the data, SPSS version 25 (IBM Corp., Armonk, NY, USA), was used. Two-way analysis of variance (ANOVA) and post hoc pairwise comparisons (Bonferroni test) were used to evaluate the axial rotation and AP translation. A paired t-test was used to evaluate the improvement in PROMs-SP. The Spearman rank correlation coefficient was used to evaluate the correlation between kinematics and PROMs-SP. The level of statistical significance was set at *p* < 0.05. A power analysis was performed with G*Power [21] using an α error of 0.05, and a 1 − β error of 0.80 to compare the means between the two groups; a sample of 44 knees was found to be sufficient for this study.

## Results

Fluoroscopic analyses were performed 13.5 ± 7.8 months after surgery. At the time of analysis, the mean age of the patients was 74.8 ± 7.2 years, the mean height was 155.0 ± 7.8 cm, and the mean body weight was 64.6 ± 10.6 kg. Of the 61 knees included in the analysis, 9 belonged to men and 52 belonged to women.

### Flexion angle and rotation angle

**Fig. 1:**
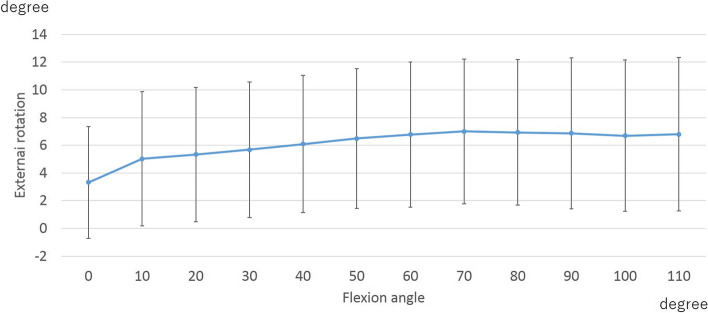
Axial rotation angle

The knees were gradually flexed from − 2.9 ± 6.4° to 109.5 ± 13.34°. In the rotation angle, the femoral component displayed an external rotation angle of 3.2 ± 3.1° relative to the tibial component from 0° to 50° of flexion. Beyond 50° of flexion, there was no significant movement (Fig. 1).

### AP translation

**Fig. 2:**
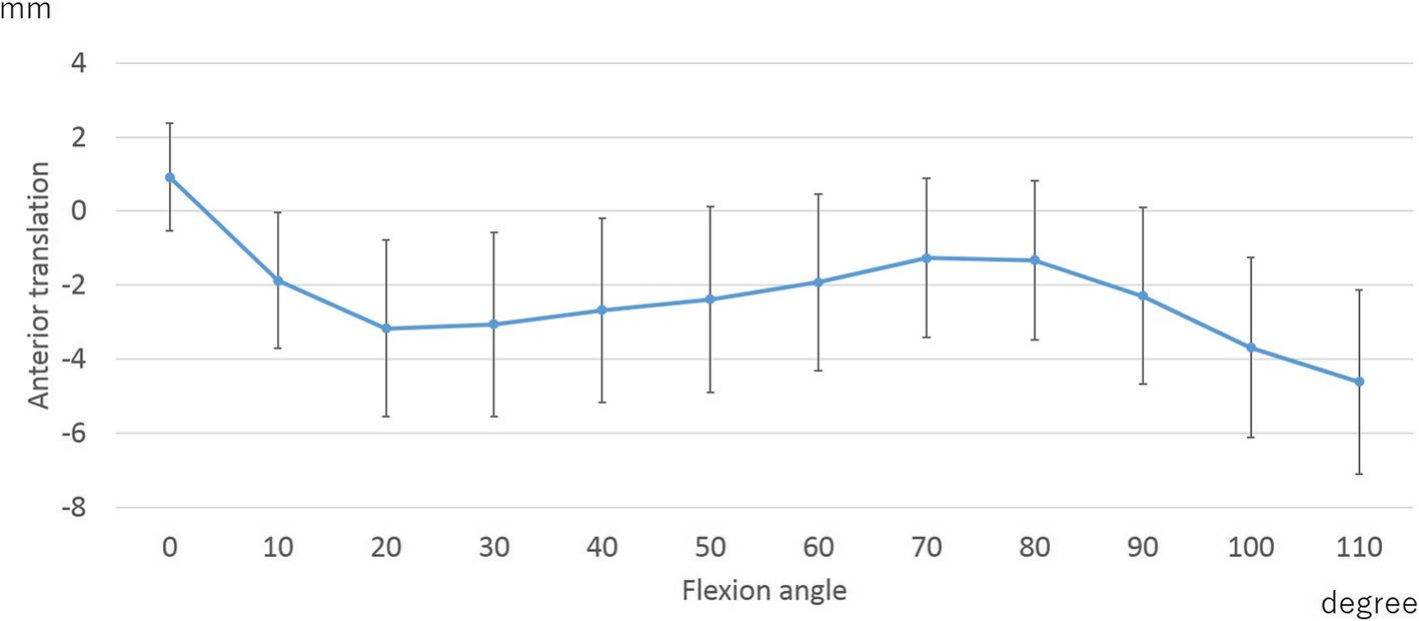
Medial anteroposterior translation

At the medial contact point, the femoral component showed 3.9 ± 2.0 mm of posterior movements from 0°–20° of flexion, followed by 1.8 ± 2.4 mm of anterior movements up to 80° of flexion. Beyond 80° of flexion, the femoral component showed 2.8 ± 1.4 mm posterior movements (Fig. 2).

**Fig. 3:**
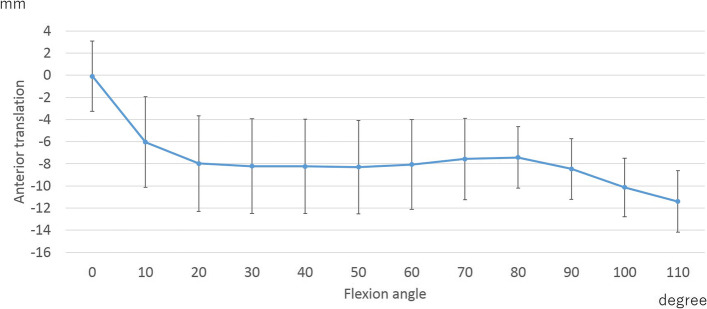
Lateral anteroposterior translation

At the lateral contact point, the femoral component showed 7.7 ± 2.8 mm of posterior movement from 0° –20° of flexion with no significant movement up to 80° of flexion. Beyond 80° of flexion, a posterior movement of 3.5 ± 1.8 mm was observed (Fig. 3).

### The improvement of PROMs-SP

As summarized in Table 1, all function, sports, and recreational activities subscales in the KOOS improved postoperatively (*p* < 0.01).

**Table 1:** The improvement of patient-reported outcome measures concerning sports (PROMs-SP)

KOOS (Function, sports and recreational activities)	Preoperative	Postoperative	Improvement
Squatting	5.0 ± 5.5	11.3 ± 6.6	6.3 ± 7.4
Running	2.6 ± 3.7	9.1 ± 6.2	6.5 ± 6.4
Jumping	2.5 ± 3.6	8.9 ± 6.1	6.4 ± 6.4
Twisting/pivoting	5.6 ± 4.8	13.9 ± 6.3	8.4 ± 7.5
Kneeling	5.0 ± 4.7	11.2 ± 6.2	6.2 ± 6.6

*KOOS* Knee Injury and Osteoarthritis Outcome Score

### Correlation between the age or gender, and PROMs-SP

The patients’ age was negatively correlated with running, and male gender was positively correlated with squatting (Table 2).

**Table 2:** The correlation between age or gender, and patient-reported outcome measures concerning sports (PROMs-SP)

	KOOS (function, sports and recreational activities)					
		Squatting	Running	Jumping	Twisting/pivoting	Kneeling
Age	rs	−0.07	−0.25	− 0.18	− 0.12	− 0.04
	*p*-value	0.57	0.046	0.15	0.34	0.72
Gender (Female = 0, Male = 1)	rs	0.29	0.07	−0.08	0.05	0.19
	*p*-value	0.03	0.57	0.52	0.71	0.13

*KOOS* Knee Injury and Osteoarthritis Outcome Score

### Correlation between the kinematics and PROMs-SP

In the rotation angle, femoral external rotation was observed from 0°-50° of flexion. The amount of femoral external rotation did not correlate with PROMs-SP (Table 3).

**Table 3:** The correlation between rotation and patient-reported outcome measures concerning sports (PROMs-SP)

Flexion angle	KOOS (Function, sports and recreational activities)					
		Squatting	Running	Jumping	Twisting/pivoting	Kneeling
0–50°	rs	0.16	0.16	0.03	0.22	0.12
	*p*-value	0.21	0.23	0.82	0.08	0.37

*KOOS* Knee Injury and Osteoarthritis Outcome Score

In medial AP translation, posterior translation was observed at 0–20° and 80–110° of flexion. Mild anterior translation was observed from 20 to 80° to flexion. Beyond 80° of flexion, posterior translation was positively correlated with squatting (Table 4).

**Table 4:** The correlation between medial anteroposterior translation and patient-reported outcome measures concerning sports (PROMs-SP)

Flexion angle	KOOS (Function, sports and recreational activities)					
		Squatting	Running	Jumping	Twisting/pivoting	Kneeling
0–20°	rs	−0.04	−0.23	− 0.22	− 0.02	0.03
	*p*-value	0.72	0.07	0.1	0.87	0.8
20–80°	rs	0.14	0.12	0.08	−0.09	−0.07
	*p*-value	0.27	0.33	0.52	0.48	0.57
80–110°	rs	0.34	0.14	0.11	0.21	0.25
	*p*-value	0.008	0.27	0.42	0.11	0.06

*KOOS* Knee Injury and Osteoarthritis Outcome Score

In lateral AP translation, posterior translation was observed from 0 to 20° and to 80–110° of flexion. Beyond 80° of flexion, posterior translation was positively correlated with running, jumping, twisting/pivoting, and kneeling (Table 5).

**Table 5:** The correlation between lateral anteroposterior translation and patient-reported outcome measures concerning sports (PROMs-SP)

Flexion angle	KOOS (Function, sports and recreational activities)					
		Squatting	Running	Jumping	Twisting/pivoting	Kneeling
0–20°	rs	−0.11	− 0.23	− 0.19	0.06	− 0.06
	*p*-value	0.36	0.07	0.15	0.64	0.67
80–110°	rs	0.16	0.42	0.3	0.34	0.28
	*p*-value	0.23	0.001	0.02	0.009	0.03

*KOOS* Knee Injury and Osteoarthritis Outcome Score

## Discussion

The most important finding of this study was that the posterior translation at high flexion was correlated with the improvements in PROMs-SP, such as squatting, running, jumping, twisting/pivoting, and kneeling. In normal knees, femoral posterior rollback is observed at high flexion [22, 23]. Kosse et al. demonstrated that sport and quality of life score were better in TKA patients with high maximal flexion (≥125°) [24]. Furthermore, Dennis et al. reported that femoral posterior movement at high flexion increased the flexion angle [25]. In addition, another study reported that the high-PROM group showed posterior femoral movement at high flexion [16]. These facts suggest that it is important to drive the femoral posterior movement at high flexion, similar to normal knees, for satisfactory sports activities after TKA. Kage et al. reported that medial and lateral AP translation did not correlate with improvements in KOOS pain, symptoms, and ADL [13]. Therefore, femoral posterior movement is required, especially during high flexion activities, for sports activities. Several studies have reported that the rate of return to sports (RTS) after TKA was > 70% [8, 26–28]. Femoral rollback-guided designs or procedures, such as a post-cam mechanism in posterior stabilized TKA, appropriate cruciate ligament tension in bicruciate-retaining TKA (BCR-TKA), or achievement of an appropriate posterior tibial slope, may improve the RTS rate. In other words, a guided motion TKA such as BCS-TKA or BCR-TKA with appropriate ligament balance might be particularly suitable for patients who are active in sports before the surgery and wish to return to work postoperatively. However, the patient age was negatively correlated with running, and male gender was positively correlated with squatting. The younger patient age may have directly influenced the superior running results, while male patients may find it easier to squat following TKA.

There was no significant correlation between rotation at early flexion and PROMs-SP in the current study. Kage et al. reported that at early flexion, femoral external rotation was positively correlated with improvement of the KOOS pain subscale [13]. These findings suggest that although after TKA, femoral external rotation and pain improves, it may be difficult to improve high flexion activities such as sports.

In this study, a mild femoral external rotation with flexion was observed. This rotational kinematic pattern was similar to that reported in previous studies [1, 13, 15, 29]. One of these previous studies reported that the lower PROM group in BCS-TKA indicated excessive femoral external rotation with flexion [15]. In the current study, the PROMs-SP improved in all subscales. Therefore, after BCS-TKA, excessive femoral external rotation may not be needed to improve sports activities.

The AP translation in this study was similar to that in previous studies [1, 13, 15, 29]. In normal knees, medial pivot motion was observed during early flexion [22]. In other words, no medial posterior translation was observed during early flexion. In contrast, the knees after BCS-TKA showed medial posterior translation during early flexion. Ishibashi et al. reported that medial posterior translation during early flexion was due to anterior post-cam engagement [29]. To achieve normal-like kinematics, further improvement of the post-cam mechanism might be required.

In this study, after BCS-TKA, all sports subscales improved. Vielgut et al. demonstrated that postoperative sports ability was correlated with preoperative activity [30]. Additionally, Ho et al. demonstrated that the RTS rate between TKA and unicompartmental knee arthroplasty did not differ under the same preoperative patient characteristics [31]. Thus, preoperative activity may have influenced the improvement of PROMs-SP.

This study had several limitations. First, in the current study, only squatting was measured. The previous studies reported that knee kinematics differ depending on the type of activities [22, 32]. Therefore, the kinematic effect during other activities might be different from squatting. Second, the squatting motion was measured only one movement repetition per patient because of ethical reasons to reduce patients’ X-ray exposure. Third, the RTS rate was not evaluated after TKA. Therefore, whether the RTS rate correlates with the PROMs-SP is unclear.

## Conclusion

Femoral rollback at high flexion during squatting may be important to improve sports performance after BCS-TKA.

## Data Availability

The datasets used during the current study are available from the corresponding author on reasonable request. The data used during the current study could not be deposited because the patients did not approve the permanent availability of data.

## Abbreviations

ADL: Activities of daily living
ANOVA: Analysis of variance
AP: Anteroposterior
BCS-TKA: Bicruciate-stabilized total knee arthroplasty
DKB: Deep knee bending
KOOS: Knee Injury and Osteoarthritis Outcome Score
PROMs: Patient-reported outcome measures
PROMs-SP: Patient-reported outcome measures concerning sports
OA: Osteoarthritis
ROM: Range of motion
RTS: Return to sports
TKA: Total knee arthroplasty
BCR-TKA: Bicruciate-retaining TKA
